# SOX17 Regulates Nestin/p16^INK4a^ Axis to Mitigate Endothelial Senescence in Pulmonary Arterial Hypertension

**DOI:** 10.21203/rs.3.rs-6999919/v1

**Published:** 2025-07-29

**Authors:** Wei Sun, Ting Wu, Zijing Zhou, Danli Jiang, Tong-you Wei, So Yun Han, John Shyy, Gang Li, Ruizheng Shi

**Affiliations:** University of California, San Diego; Xiangya Hospital, Central South University; Xiangya Hospital, Central South University; Hainan Medical University; University of California, San Diego; University of California, San Diego; University of California; University of Pittsburgh; Xiangya Hospital, Central South University

**Keywords:** Nestin, SOX17, Cyclin-dependent kinase inhibitor 2A, Endothelial cell senescence, Pulmonary arterial hypertension

## Abstract

Emerging evidence indicates that endothelial cell senescence plays a critical role in the pathogenesis of pulmonary arterial hypertension (PAH). However, the underlying mechanisms and signaling pathways driving pulmonary endothelial senescence in PAH remain incompletely understood. In this study, we used a novel functional genomics approach to show that the intermediate filament protein Nestin binds to a *cis*-regulatory element (*cis*-RE) on the *cyclin*-*dependent kinase inhibitor 2A/B (CDKN2A/B)* locus, repressing p16^INK4a^ expression and mitigating cellular senescence in human pulmonary arterial endothelial cells (PAECs). Consistently, Nestin expression was markedly downregulated in both PAH patients and rodent models, leading to increased p16^INK4a^ level and enhanced endothelial senescence in PAH-affected lungs. We further demonstrated that SRY-related HMG-box 17 (SOX17), a transcription factor known to be associated with PAH, activated Nestin expression by binding directly to the Nestin promoter, which inhibited cellular senescence by suppressing p16^INK4a^ expression in PAECs. In vivo, SOX17 overexpression, which leads to upregulation of Nestin and downregulation of p16^INK4a^ in lungs of PAH rat models, significantly reduced PAEC senescence, attenuated pulmonary vascular remodeling, and alleviated PAH severity. Conversely, silencing of Nestin in the SOX17 overexpressing PAECs exacerbated PAEC senescence and worsened PAH in rodents. Our findings reveal a novel SOX17–Nestin–p16^INK4a^ regulatory pathway that governs pulmonary endothelial cell senescence, which offers new insights into PAH pathobiology and represents a promising therapeutic target for intervention.

## Introduction

Pulmonary hypertension (PH) is defined by mean pulmonary artery pressure (mPAP) > 20 mmHg at rest, as measured by right heart catheterization^1^. A severe subset of PH, pulmonary arterial hypertension (PAH), is characterized by progressive pulmonary vascular remodeling, leading to significantly increased pulmonary vascular resistance and elevated mPAP^2^. If untreated, PAH progresses to right ventricular failure and death^3^. Despite current treatments, PAH remains a life-threatening disease with poor prognosis, as evidenced by reported 5-year survival rates of approximately 60–65%^4,5^. This poor prognosis is largely attributed to an incomplete understanding of the molecular mechanisms driving PAH pathogenesis.

Endothelial dysfunction is a key contributor to the pathogenesis of PAH^6^. Pulmonary artery endothelial cells (PAECs) undergo multiple dynamic and pathological changes throughout both the early and progressive stages of the disease^7–9^. Among these alterations, PAEC senescence has gained increasing attention as a factor contributing to PAH^10–12^. Cellular senescence is a state of irreversible cell cycle arrest accompanied by chromatin remodeling and the secretion of pro-inflammatory and profibrotic factors, collectively termed the senescence-associated secretory phenotype (SASP)^13^. While SASP can promote the proliferation of smooth muscle cells (SMCs), contributing to vascular remodeling, SASP can also induce secondary senescence in neighboring ECs, further perpetuating the progression of PAH^12,14^. Genome-wide association study has identified an association of aging and age-related disease with the *cyclin*-*dependent kinase inhibitor 2A/B (CDKN2A/B)* locus^15,16^, a locus that contains three tumor suppressor genes—p14^ARF^, p15^INK4b^, and p16^INK4a^—as well as a long noncoding RNA, antisense noncoding RNA in the INK4 locus (ANRIL). Among these genes, p16^INK4a^ is a marker and an activator of cellular senescence^17,18^. The accumulation of p16^INK4a^-positive cells is linked to aging-related organ dysfunction, and the clearance of these maladapted cells delays the onset of age-related diseases and extends the lifespan in mice^19,20^. In relation to PAH, p16^INK4a^ expression has been found elevated in pulmonary arterial ECs (PAECs) in diseased vessels^10^. However, how PAH-related p16^INK4a^ expression is regulated in PAH pathogenesis remains unexplored.

Nestin, a class VI intermediate filament protein, is a well-established marker of progenitor and stem cells^21,22^. Emerging evidence implicates Nestin in vascular remodeling processes, including PAH^23–25^. However, the cell-specific expression of Nestin in PAH remains controversial. Saboor et al. reported that Nestin is absent in PAECs under both normal and PAH conditions but is highly expressed in proliferating pulmonary artery SMCs^23^. In contrast, Bhagwani et al. identified PAECs as a significant source of Nestin expression in PAH^24^.

SOX17 is a transcription factor belonging to the SOX (SRY-related HMG-box) family, essential for EC identity and vascular development^26^. Previous studies have established that SOX17 is downregulated in PAH, leading to EC dysfunction and aberrant vascular remodeling^27–30^. Although it was reported that SOX17 can control the emergence and remodeling of Nestin-expressing coronary vessels during cardiac development^31^, it remains unknown if SOX17 plays a role in regulating Nestin endogenous expression in the vascular remodeling under pathological conditions.

Previously, by coupling high-throughput mapping with proteomics analysis, we identified S1606^18^, a *cis*-regulatory element (*cis*-RE) at the *CDKN2A/B* locus, that can regulate p16^INK4a^ expression via transcription factor binding, thus contributing to cellular senescence. However, whether S1606-mediated p16^INK4a^ regulation participates in endothelial senescence during PAH pathogenesis is unclear. In this study, by applying flanking restriction-enhanced DNA pulldown mass spectrometry (FREP-MS), a novel technique developed in our laboratory^18^, we discovered that Nestin is a regulatory protein that suppresses cellular senescence by downregulating p16^INK4a^ via its binding to the *cis*-RE S1606 on the *CDKN2A/B* locus. Importantly, we confirmed that Nestin expression is significantly downregulated in PAECs from both PAH patients and rodent models of experimental PAH. We also identified that SOX17 is a transcription activator of Nestin as overexpression of SOX17 results in upregulation of Nestin and downregulation of p16^INK4a^, thereby mitigating endothelial senescence. Consistently, we showed that, in PAH, the downregulation of SOX17 can lead to upregulation of p16^INK4a^ and p16^INK4a^ -dependent endothelial senescence by suppressing the expression of Nestin. Our study indicates that the impaired SOX17–Nestin axis in pulmonary ECs promotes a senescent phenotype that aggravates PAH.

## Results

### Identification of Nestin specifically binding to *cis*-RE S1606

**Figure 1:**
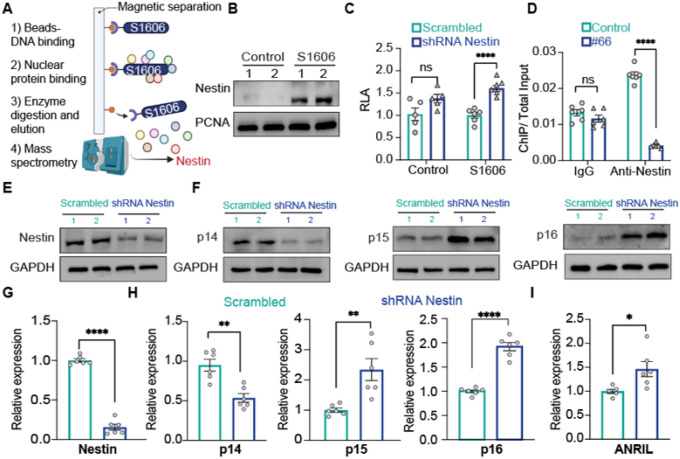
Identification of Nestin-specific binding to S1606. A. Identification of Nestin binding to S1606 by FREP-MS; B. DNA pull-down western blot assay demonstrating that Nestin binds to S1606; C. Luciferase reporter assay indicating that Nestin binds to S1606 and regulates gene transcription in HEK293 cells; D. ChIP assay showing binding of Nestin to S1606; E-H. Western blot assay (E, F) and qPCR (G, H) analysis Nestin, p14^ARF^, p15^INK4b^, p16^INK4a^ expression after Nestin downregulation. I. qPCR analysis of antisense noncoding RNA in the INK4 locus (ANRIL) expression after Nestin downregulation; n=3 or 6; *: p<0.05; **: p<0.01; ***: p<0.001; ****: p<0.0001; ns: not significant; scale bar: 100 μm; Sequence of S1606: GGAAATGTGATCTTAAAATTATAGGACCTCAAATT; sequence of #66 clone: GGAAATGTGATCTTAAAATT**T**ATAGGACCTCAAATT/ GGAAATGTGATCTTAAAATTATAGG**G**CCTCAAATT.

Using a high-throughput mapping and proteomics approach, we previously identified a *cis*-RE named S1606 in the *CDKN2A/B* locus that regulated p16^INK4a^- and p16^INK4a^-dependent cellular senescence^16,18^. To further explore the role of this *cis*-RE, we applied FREP-MS (Fig. 1A) using *cis*-RE S1606 as a “bait” to pull down regulatory protein(s) in nuclear extracts from human ECs and identified Nestin as a candidate protein. The binding of Nestin to S1606 was first confirmed by DNA pull-down–Western blot analysis, showing specific attachment of Nestin to S1606 (Fig. 1B) and by a conditioned luciferase reporter assay, demonstrating significantly increased reporter activity driven by the S1606 sequence in shRNA-mediated Nestin knockdown in HEK293 cells versus wild-type (WT) control cells (Fig. 1C). Next, we performed a chromatin immunoprecipitation (ChIP) assay in cells with the S1606 sequence mutated by CRISPR-Cas9–mediated mutagenesis and demonstrated an endogenous binding of Nestin to S1606 by showing a reduced binding of Nestin to S1606 in the S1606-mutated cells (clone #66) as compared with WT control cells (Fig. 1D). Together, our data demonstrate a specific binding of Nestin to S1606 on the CDKN2A/B locus.

### Nestin regulates p14^ARF^, p15^INK4b^, p16^INK4a^, and ANRIL expression in PAECs

Nestin binding to S1606 on the *CDKN2A/B* locus suggested that Nestin might be a transcriptional regulator controlling the expression of p14^ARF,^ p15^INK4b^, p16^INK4a^, and the noncoding RNA ANRIL on this locus. To demonstrate this, we downregulated Nestin in PAECs by shRNA-mediated knockdown, which resulted in significantly increased both protein and mRNA levels of p15^INK4b^ and p16^INK4a^ but a decreased level of p14^ARF^ (Fig. 1E–1H). The expression of the long non-coding RNA ANRIL was increased in PAECs with shRNA-mediated Nestin knockdown (Fig. 1I). These results were further confirmed by siRNA-mediated knockdown of Nestin with an RNAi targeting sequence different from that used in the shRNA-mediated knockdown (**Supplementary Fig. 1A**). Conversely, ectopic overexpression of Nestin in PAECs decreased the levels of p15^INK4b^, p16^INK4a^, and ANRIL but increased p14^ARF^ level (**Supplementary Fig. 1B** and **1C**).

These data, together with data in Fig. 1B–1D, demonstrate that Nestin is a transcriptional regulator that can modulate the expression of p14^ARF^, p15^INK4b,^ p16^INK4a^ and ANRIL within the *CDKN2A/B* locus by binding to the *cis*-RE S1606 in PAECs.

### Nestin suppresses cellular senescence by downregulating p16^INK4a^

**Figure 2:**
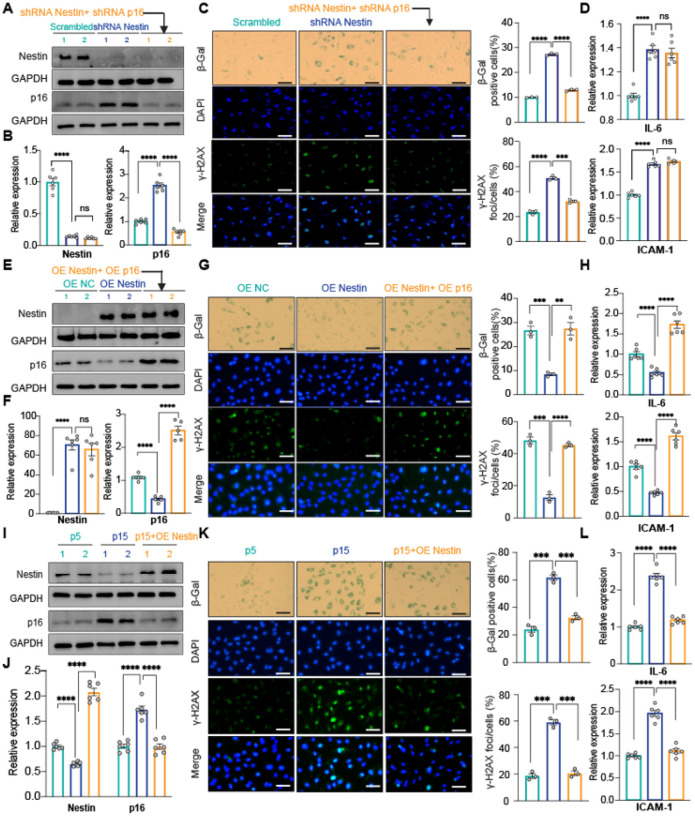
Nestin suppresses cellular senescence by downregulating p16^INK4a^ expression *Scrambled, Nestin*- *and/or p16*^*INK4a*^-*knockdown PAECs:* A. Western blot assay and B. qPCR assay of Nestin and p16^INK4a^ expression; C. SA-β-gal and γ-H2AX staining of β-gal and γ-H2AX expression; D. qPCR of SASP gene expression; *Scrambled, Nestin*- *and/or p16*^*INK4a*^-*overexpression PAECs:* E. Western blot assay and F. qPCR of Nestin and p16^INK4a^ expression; G-H. SA-β-gal and γ-H2AX staining (G) and expression of SASP genes (H). *Early passage (p5), late passage (p15) and Nestin*-*overexpressed p15 PAECs:* I. Western blot assay and J. qPCR of Nestin and p16^INK4a^ expression; K-L. SA-β-gal and γ-H2AX staining (K) and expression of SASP genes (L). n=3 or 6; *: p<0.05; **: p<0.01; ***: p<0.001; ****: p<0.0001; ns: not significant; scale bar: 100 μm.

Because p16^INK4a^ is a marker and activator of cellular senescence^32^, activation of p16^INK4a^ by Nestin suggests that Nestin could be an inducer of cellular senescence in PAECs. To test this, we performed functional complementation assays to evaluate cellular senescence in PAECs with shRNA-mediated Nestin knockdown or PAECs with Nestin and p16^INK4a^ double knockdown. Knockdown of Nestin in PAECs recapitulated an upregulated p16^INK4a^ expression (Fig. 2A, **left and middle two lanes, and** 2B) and induced cellular senescence as evidenced by both increased SA-β-gal and γ-H2AX staining (Fig. 2C, **left and middle column**), and upregulated expression of the SASP genes interleukin 6 (IL-6) and intercellular adhesion molecule-1 (ICAM-1) (Fig. 2D, **left and middle lane**). However, in PAECs with Nestin and p16^INK4a^ double knockdown (Fig. 2A, **right lane**), cellular senescence was suppressed as measured by decreased SA-β-gal and γ-H2AX staining (Fig. 2B, **right lane;** Fig. 2C, **right lane**). Notably, IL-6 and ICAM-1 levels remained unchanged in PAECs with Nestin and p16^INK4a^ double knockdown (Fig. 2D, **middle and right lane**), which is consistent with previous publications demonstrating that p16^INK4a^ is not a regulator of SASP genes^33^. We also checked cell cycle arrest as another marker for cellular senescence and a significantly decreased number of cells in the S/G2/M phase in Nestin-downregulated cells was observed. Consistently, p16^INK4a^ and Nestin double knockdown increased the proportion of cells in the S/G2/M phase (**Supplementary Fig. 2**). Conversely, in Nestin-overexpressing PAECs, p16^INK4a^ was inactivated (Fig. 2E, **left and middle two lanes, and 2F**) and cellular senescence was repressed (Fig. 2G, **left and middle column and** Fig. 2H, **left and middle lane**); overexpression of p16^INK4a^ in Nestin-overexpressing PAECs completely restored cellular senescence (Fig. 2E, **right two lanes;** Fig. 2G, **right column;** Fig. 2H, **right lane**).

We previously demonstrated an involvement of p16^INK4a^ in replicative senescence in human aortic ECs by showing a passage-dependent upregulation of p16^INK4a^ in late-passage aortic ECs^16^. Consistent with the role of Nestin as a p16^INK4a^ repressor, we also detected a passage-dependent downregulation of Nestin, along with a passage-dependent upregulation of p16^INK4a^, in late passage (p15) versus early passage (p5) human PAECs (Fig. 2I, **left and middle two lanes,** and 2J). Consistently, in p15 PAECs, cellular senescence was upregulated, as evidenced by increased SA-β-gal and γ-H2AX staining and upregulated levels of SASP genes IL-6 and ICAM-1 (Fig. 2K and 2L, **left and middle**). As expected, overexpression of Nestin in p15 PAECs restored p16^INK4a^ expression as well as cellular senescence (Fig. 2I–2L, **right lanes**). These data suggest a role for Nestin in replicative senescence.

Taken together, our data demonstrate that Nestin is a repressor of cellular senescence, inhibiting p16^INK4a^-dependent cellular senescence in human PAECs.

### Decreased SOX17–Nestin axis and elevated p16^INK4a^ expression in PAH

**Figure 3:**
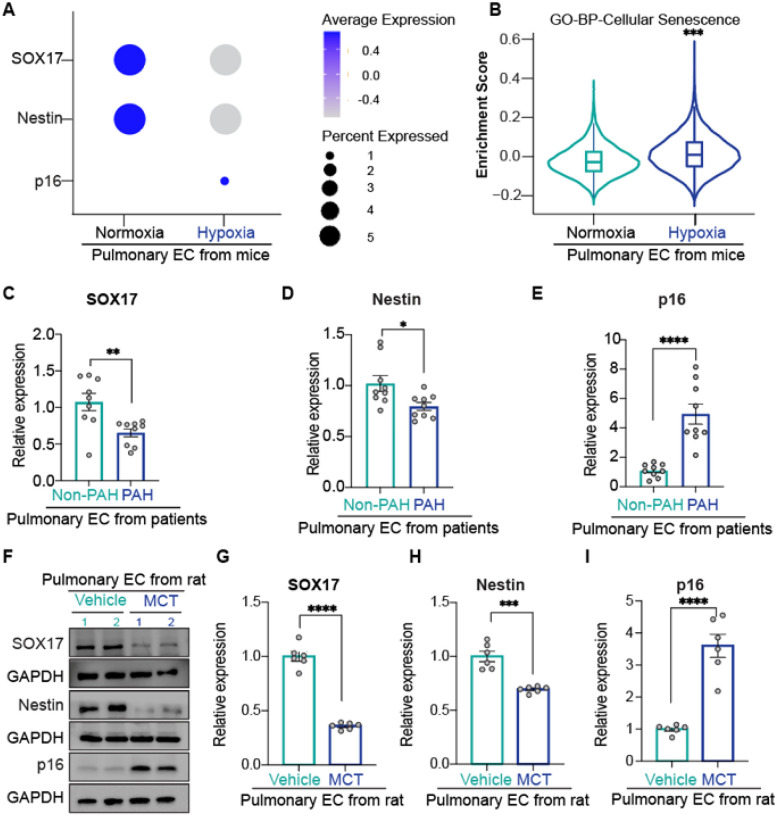
Decreased SOX17–Nestin axis and elevated p16^INK4a^ expression in PAH. A. The expression patterns of SOX17, Nestin, and p16^INK4a^ in murine endothelial cells (ECs) under pulmonary hypertension conditions or not characterized by using single-cell RNA sequencing data from the GSE154959 dataset. B. Enrichment scores for cellular senescence pathways in PAH ECs from the GSE154959 dataset; C-E. qPCR assay demonstrating SOX17 (C), Nestin (D), and p16^INK4a^ (E) expression in ECs from PAH patients and non-PAH patients; F-I. Western blot (F) and qPCR (G-I) assay of SOX17, Nestin and p16^INK4a^ expression in PAH rat ECs and controls with and without monocrotaline (MCT) treatment; n=3 or 6; *: p<0.05; **: p<0.01; ***: p<0.001; ****: p<0.0001.

Regulation of p16^INK4a^ -dependent cellular senescence by Nestin in human PAECs suggests a role for Nestin-mediated senescence in pulmonary vascular remodeling in PAH. To explore this, we first analyzed data obtained from the single-cell RNA sequencing (scRNA-seq) dataset GSE154959, which profiles murine ECs in the setting of PAH^34^. Consistently, this analysis revealed downregulated Nestin and upregulated p16^INK4a^ in PAH ECs as compared with control cells (Fig. 3A). Moreover, Gene Ontology (GO) analysis of PAH ECs revealed an enriched score for the biological cellular senescence pathway (Fig. 3B). These data suggest that Nestin might play a role in PAH-associated EC senescence *in vivo*. Using the same scRNA-seq dataset, we also identified a downregulation of SOX17 in PAH ECs (Fig. 3A). Although previous studies established an indispensable role for SOX17 in PAH pathogenesis^35^, the interplay between SOX17 and Nestin-mediated cellular senescence remained unexplored. To validate these findings in human PAH, our qPCR analysis demonstrated a significantly reduced expression of both SOX17 and Nestin, with concomitant elevated level of p16^INK4a^ in pulmonary microvascular ECs from PAH patients versus non-diseased ECs (Fig. 3C–E). Western blot and qPCR analyses also demonstrated downregulated expression of both SOX17 and Nestin, along with upregulated p16^INK4a^ expression in pulmonary microvascular ECs from rats with PAH induced by monocrotaline (MCT) as compared with control ECs (Fig. 3F–I).

Together, these results suggest that SOX17 could be a transcriptional activator of Nestin, inducing p16^INK4a^-dependent endothelial senescence in the development of PAH.

### SOX17 is a transcriptional activator of Nestin

**Figure 4:**
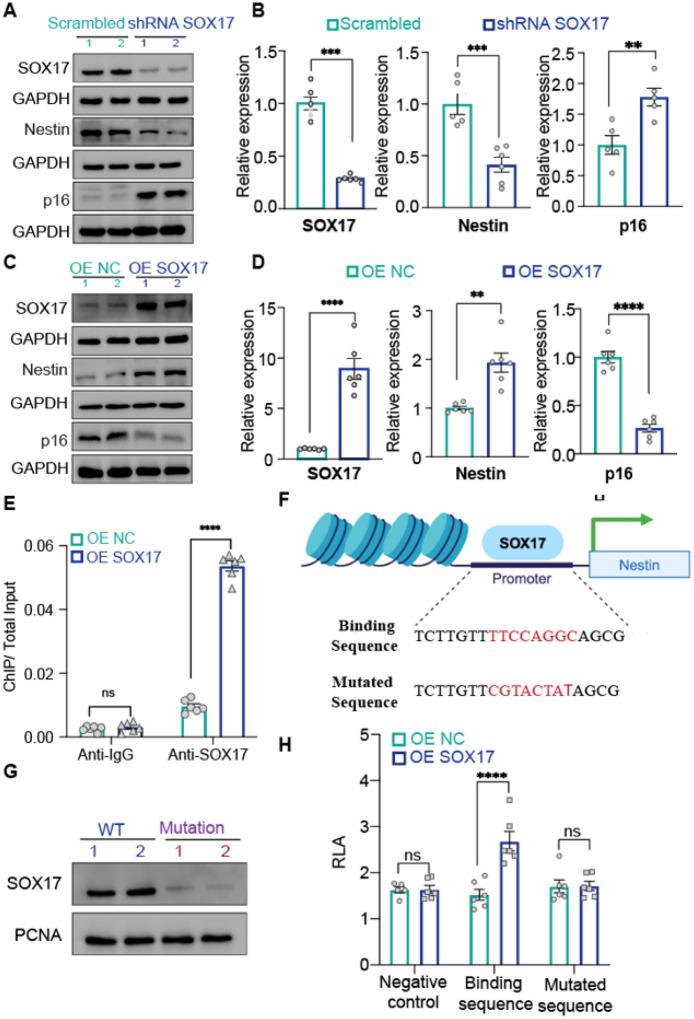
SOX17 is a transcriptional activator of Nestin. A. Westen blot and B. qPCR assay of SOX17, Nestin and p16^INK4a^ expression in SOX17-downregulated pulmonary arterial endothelial cells (PAECs); C. Westen blot and D. qPCR assay of SOX17, Nestin and p16^INK4a^ expression in SOX17-overexpressed PAECs; E. ChIP showing the specific binding of SOX17 with sequence (TTCCAGGC) within the Nestin promoter; F. Binding sequence and mutated binding sequence. G. DNA pull-down western blot assay demonstrating that SOX17 binds to the sequence; H. Luciferase reporter assay indicating that SOX17 binds to the sequence within the Nestin promoter region; n=3 or 6; *: p<0.05; **: p<0.01; ***: p<0.001; ****: p<0.0001.

To demonstrate that SOX17 regulates Nestin, we perturbed SOX17 in PAECs. ShRNA-mediated SOX17 knockdown resulted in downregulated Nestin with concurrent upregulated p16^INK4a^ at both the protein and mRNA levels (Fig. 4A and 4B). Conversely, ectopic overexpression of SOX17 increased Nestin level but decreased p16^INK4a^ level (Fig. 4C and 4D). To further demonstrate that SOX17 is a transcriptional regulator of Nestin, we used six putative SOX17 binding sequences identified within the 2-kb Nestin promoter region. ChIP assay revealed that SOX17 preferentially binds to the sequence of TTCCAGGC located in ~500 bp upstream of the Nestin transcription start site (Fig. 4E, and 4F; **Supplementary Fig. 3**). This specific binding was validated by DNA pulldown assay with the WT SOX17 binding sequence versus the mutated CGTACTATAC sequence, showing a decreased SOX17 protein level pulled down by this mutated sequence (Fig. 4F and 4G). Additionally, the specific binding was verified by a conditional luciferase reporter assay with a reporter construct that carries an irrelevant sequence as a negative control, the SOX17 binding sequence TTCCAGGC or the mutated sequence CGTACTATAC. Overexpression of SOX17 significantly increased luciferase reporter activity in cells transfected with the reporter construct containing the SOX17 binding sequence, with no obvious change in cells transfected with the constructs containing neither the irrelevant sequence nor the mutated sequence (Fig. 4H). Together, these results demonstrate that SOX17 is a transcriptional activator of Nestin as SOX17 can upregulate Nestin by binding to a SOX17 binding site identified on the Nestin promoter region.

### SOX17 inhibits cellular senescence by inducing Nestin expression

**Figure 5:**
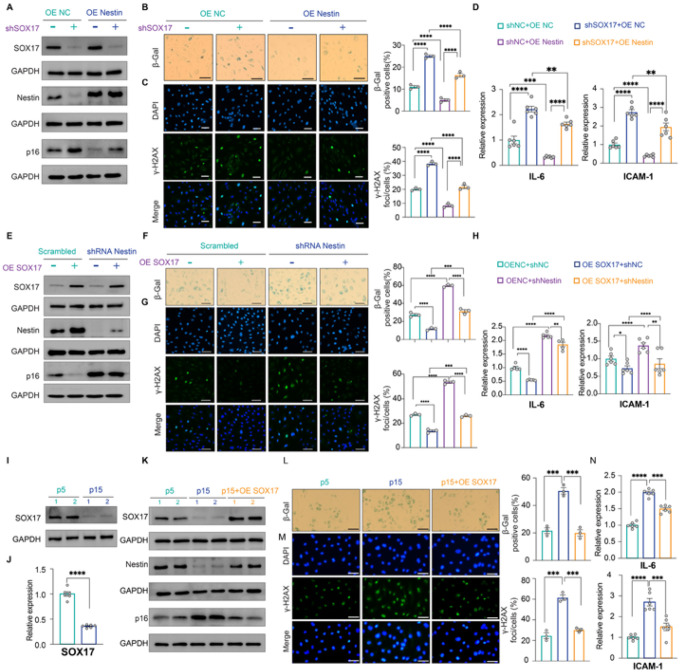
SOX17 inhibits cellular senescence by inducing Nestin expression. *Scrambled, SOX17*-*downregulated and/or Nestin*-*overexpressed PAECs:* A. Western blot assay of SOX17, Nestin and p16^INK4a^ expression; B. SA-β-gal, C. γ-H2AX staining and D. expression of SASP genes. *Scrambled, SOX17*-*overexpression and/or Nestin*-*downregulated PAECs*: E. Western blot assay of SOX17, Nestin and p16^INK4a^ expression; F. SA-β-gal, G. γ-H2AX staining and H. the expression of SASP genes. *P5 and p15 PAECs*: I. Western blot assay and J. qPCR analysis of SOX17 expression. *P5, p15 and SOX17*-*overexpressed p15 PAECs*: K. Western blot assay of SOX17, Nestin and p16^INK4a^ expression. L. SA-β-gal, M. γ-H2AX staining and N. expression of SASP genes. n=3 or 6; *: p<0.05; **: p<0.01; ***: p<0.001; ****: p<0.0001; ns: not significant; scale bar: 100 μm.

To investigate whether SOX17 regulates cellular senescence by modulating p16^INK4a^ expression through Nestin, we investigated cellular senescence in PAECs with shRNA-mediated SOX17 knockdown. Downregulation of SOX17 as well as Nestin and upregulation of p16^INK4a^ were first confirmed by western blot analysis (Fig. 5A, **lanes 1 and 2**) and qPCR analysis (**Supplementary Fig. 4**). Consistent with the increased expression of p16^INK4a^, SOX17 knockdown increased the level of cellular senescence as measured by both SA-β-gal and γ-H2AX staining (Fig. 5B and 5C, **columns 1 and 2**) as well as expression of the SASP genes IL-6 and ICAM-1 (Fig. 5D, **lanes 1 and 2**). However, as expected, overexpression of Nestin in PAECs with shRNA-mediated SOX17 knockdown, which downregulated p16^INK4a^ expression (Fig. 5A, **lanes 2 and 4**), overrode the senescent phenotype induced by SOX17 knockdown, as evidenced by reduced SA-β-gal activity, decreased γ-H2AX staining, and diminished SASP gene expression (Fig. 5B and 5C, **columns 2 and 4;** Fig. 5D, **lanes 2 and 4**). Conversely, overexpression of SOX17, which activated Nestin and inhibited p16^INK4a^ (Fig. 5E, **lanes 1 and 2**), suppressed cellular senescence (Fig. 5F and 5G, **columns 1 and 2;** Fig. 5H, **lanes 1 and 2**), and shRNA-mediated Nestin knockdown in SOX17-overexpressing PAECs, which upregulated p16^INK4a^ (Fig. 5E, **lanes 2 and 4**), induced cellular senescence (Fig. 5F and 5G, **columns 2 and 4;** Fig. 5H, **lanes 2 and 4**).

We further investigated whether SOX17 plays a role in replicative senescence. Consistent with the downregulation of Nestin and upregulation of p16^INK4a^ in p15 versus p5 PAECs (Fig. 2I and 2J), SOX17 level was decreased in p15 versus p5 PAECs (Fig. 5I and 5J). However, overexpression of SOX17 in p15 PAECs, which upregulated Nestin and downregulated p16^INK4a^ (Fig. 5K, **middle and right two lanes**), reduced cellular senescence, as evidenced by both SA-β-gal and γ-H2AX staining (Fig. 5L and 5M, **columns 2 and 3**), as well as expression of the SASP genes IL-6 and ICAM-1 (Fig. 5N, **lanes 2 and 3**).

Taken together, these results demonstrate that SOX17 is a repressor of cellular senescence at least in PAECs, upregulation of SOX17 inhibits p16^INK4a^-dependent cellular senescence by activating Nestin.

### SOX17–Nestin axis regulates PAEC senescence under PAH-relevant stimuli

**Figure 6:**
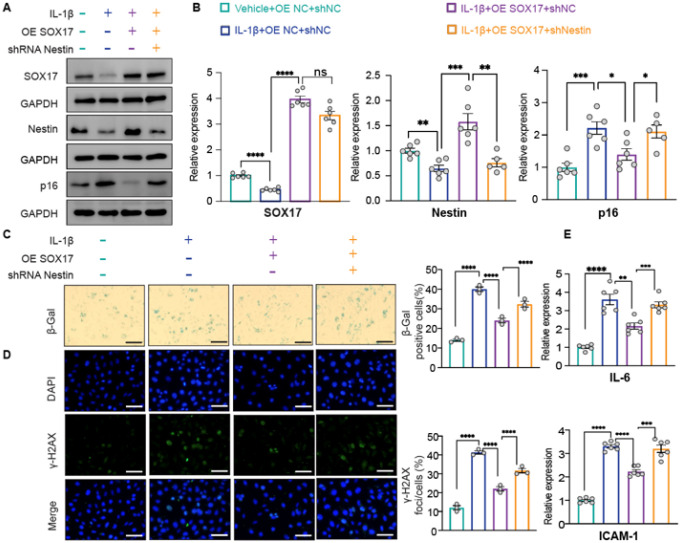
SOX17/Nestin regulates endothelial senescence in PAH pathological stimuli. *Control PAECs and PAECs that were treated with IL*-*1β or treated with IL*-*1β and overexpressed SOX17 and/or downregulated Nestin:* A. Western blot assay and B. qPCR analysis of SOX17, Nestin and p16^INK4a^ expression; C. SA-β-gal, D. γ-H2AX staining and E. expression of SASP genes. n=3 or 6; *: p<0.05; **: p<0.01; ***: p<0.001; ****: p<0.0001; ns: not significant; scale bar: 100 μm.

IL-1β, a proinflammatory cytokine known to induce a PAH-like EC phenotype, downregulated both SOX17 and Nestin and upregulated p16^INK4a^ in PAECs (Fig. 6A and 6B, **lanes 1 and 2**), subsequently inducing EC senescence (Fig. 6C and 6D, **columns 1 and 2**). SOX17 overexpression suppressed p16^INK4a^ and senescence phenotypes, and Nestin knockdown eliminated this protective effect, thus suggesting that the SOX17–Nestin–p16^INK4a^ axis mediated the IL-1β–induced senescence (Fig. 6C–6E, **columns 3 and 4**). Notably, these senescence-associated changes were associated with impaired endothelial tube formation capacity, which was restored by SOX17 overexpression but attenuated by Nestin knockdown (**Supplementary Fig. 5**). These findings were corroborated in a hypoxia-induced PAH model: hypoxic ECs isolated from mice showed decreased SOX17/Nestin level and increased p16^INK4a^ level with enhanced senescence (**Supplementary Fig. 6A** and **6B**). Notably, SOX17 overexpression rescued Nestin expression and attenuated senescence markers, effects that were reversed by Nestin knockdown (**Supplementary Fig. 6**).

These findings demonstrate that the SOX17–Nestin axis antagonized EC senescence under PAH-relevant stimuli.

### SOX17–Nestin axis attenuates MCT-induced PAH

**Figure 7:**
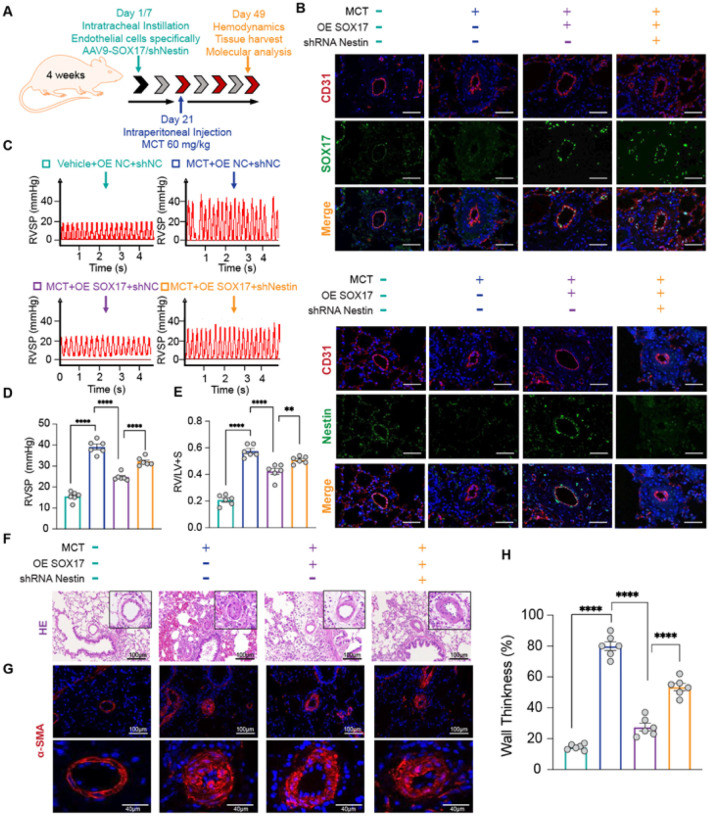
SOX17–Nestin axis attenuates MCT-induced PAH in rats. A. Endothelial-specific adeno-associated virus (AAV)-based approach by intratracheal injection to overexpress SOX17 and knock down Nestin in rats. Then, rats were subjected to 4-week MCT treatment to induce PAH; B. Immunofluorescent staining of the distribution and expression of SOX17 and Nestin; C. Representative tracing; D. quantification of right ventricular systolic pressure (RVSP); E. Ratio of right ventricle to left ventricle plus septum weight; F. Representative images of hematoxylin-eosin staining of pulmonary arteries; G. Representative images of α-SMA immunostaining of pulmonary arteries; H. Quantification of pulmonary artery thickness measured by the ratio of vessel wall area to total vessel area. n=3 or 6; n=3 or 6; *: p<0.05; **: p<0.01; ***: p<0.001; ****: p<0.0001; ns: not significant.

**Figure 8:**
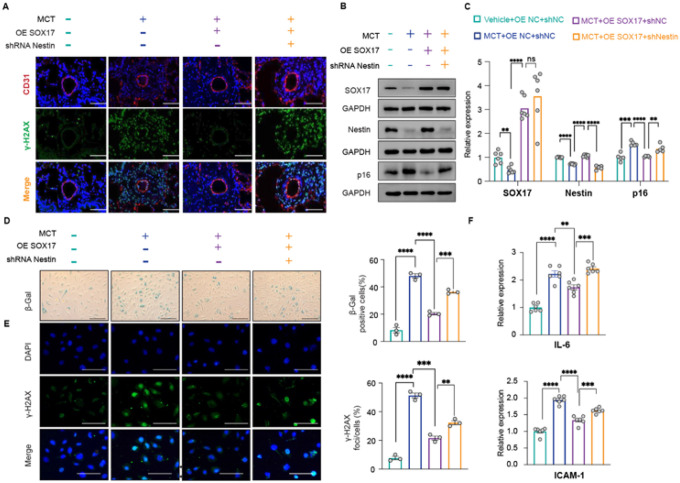
SOX17–Nestin axis attenuates MCT-induced PAH in rats by inhibiting endothelial senescence. *Primary pulmonary rat ECs treated with vehicle, MCT, and MCT with overexpressed SOX17 and/or downregulated Nestin:* A. Immunofluorescent staining of the distribution and expression of γ-H2AX; B. Western blot assay and C. qPCR analysis of SOX17, Nestin and p16^INK4a^ expression; D. SA-β-gal, E. γ-H2AX staining and F. expression of SASP genes. n=3 or 6; *: p<0.05; **: p<0.01; ***: p<0.001; ****: p<0.0001; ns: not significant; scale bar: 100 μm.

To investigate the effects of the SOX17–Nestin axis on PAH *in vivo*, we used an EC-specific adeno-associated virus (AAV) vector to overexpress SOX17 or downregulate Nestin in MCT-treated rats (Fig. 7A). Immunofluorescence staining verified decreased SOX17 and Nestin levels in rats with PAH induced by MCT (Fig. 7B). SOX17 overexpression significantly reduced PH severity, as evidenced by lower right ventricular systolic pressure (RVSP) and reduced right ventricular hypertrophy (Fig. 7C–7E), consistent with the recovered Nestin levels in lungs (Fig. 7B, **columns 2 and 3**). Hematoxylin-eosin staining and α-SMA immunofluorescence demonstrated that SOX17 overexpression markedly attenuated pulmonary vascular remodeling in MCT-treated rats (Fig. 7F–7H, columns 2 and 3). Importantly, Nestin knockdown concomitantly diminished the protective effects of SOX17 overexpression and regained the PAH pathology (Fig. 7B–7H, **column 3 and 4**). To confirm the EC senescence involved in PAH, we verified the elevated level of γ-H2AX in lung tissues from PAH rats (Fig. 8A). This elevated γ-H2AX level was suppressed by SOX17 overexpression (Fig. 8A, **columns 2 and 3**) and further reemerged with Nestin knockdown in SOX17-overexpressing PAH rats (Fig. 8A, **columns 3 and 4**). In line with these animal experiments, pulmonary ECs from these rats showed corresponding changes in SOX17, Nestin, and p16^INK4a^ levels (Fig. 8B and 8C). ECs from PAH rats showed elevated p16^INK4a^ expression and increased levels of cellular senescence markers (SA-β-gal and γ-H2AX, SASP factors) (Fig. 8D and 8E, **columns 1 and 2;** Fig. 8F, **lanes 1 and 2**), whereas SOX17 overexpression ameliorated and Nestin knockdown restored these senescence-related cellular events (Fig. 8D and 8E, **column 3 and 4;** Fig. 8F, **lane 3 and 4**).

Collectively, these findings demonstrate that the SOX17–Nestin axis may play a crucial role in PAH pathogenesis by inducing EC senescence.

## Discussion

**Figure 9:**
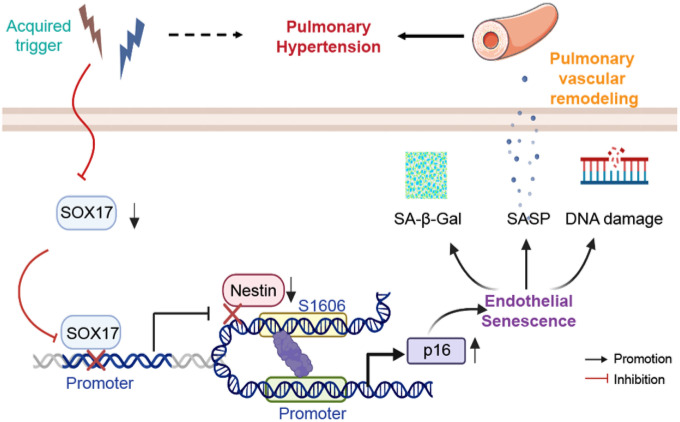
Schematic illustration of SOX17 regulating Nestin–p16^INK4a^ axis to mediate pulmonary EC senescence under PAH conditions

In this report, we reveal a novel SOX17–Nestin–p16^INK4a^ regulatory axis that protects PAECs against senescence. SOX17 directly bound the Nestin promoter to upregulate its expression, which in turn suppressed p16^INK4a^ transcription by binding to a specific *cis*-RE (S1606) in the *CDKN2A/B* locus. Our findings reveal that loss of SOX17 and Nestin in PAECs drove cellular senescence in PAH, with maintenance of this axis being essential for endothelial homeostasis. Given the established role of EC senescence in PAH pathogenesis^12,14,36,37^, our study provides a mechanistic explanation for how the SOX17–Nestin axis contributes to vascular remodeling (Fig. 9).

Our findings extend previous work on SOX17, a gene previously implicated in heritable PAH^27,38,39^. Prior work showed the importance of SOX17 for normal pulmonary endothelial function^40,41^, with rare SOX17 variants conferring increased PAH risk and endothelial SOX17 deficiency exacerbating PAH^29,30,35,42,43^. Consistent with these reports, we demonstrated that SOX17 overexpression in the pulmonary endothelium attenuated MCT-induced PAH in rats. Previous studies linked SOX17 loss to aberrant growth factor signaling and metabolism in PAH^28,30,44^, but we have identified cellular senescence as a previously unrecognized consequence of SOX17 deficiency. Additionally, we provide the first evidence of a functional interaction between SOX17 and Nestin in PAECs under pathologic conditions. Although SOX17 was shown to regulate a Nestin enhancer during embryonic coronary vessel formation^31^, we now demonstrate that in the adult lung, SOX17 directly drives Nestin endogenous expression under pathologic stress.

Previous studies of Nestin in PAH showed contradictory findings on its expression in vascular cells. Saboor et al.^23^ reported undetectable Nestin in PAECs, whereas Bhagwani et al.^24^ demonstrated Nestin-positive ECs in PAH vascular lesions. Our results reconcile these findings by confirming that PAECs express Nestin, although at relatively low levels under control conditions and further decreased in PAH models. This finding explains why detection might be difficult with less sensitive methods such as the immunofluorescence used by Saboor et al^23^. Although Bhagwani et al^24^ observed increased Nestin level in specific endothelial subpopulations in complex lesions, our findings suggest that this represents an adaptive response in certain ECs rather than a universal feature. The authors’ observation that Nestin overexpression enhances EC proliferation aligns with our finding that it suppresses cellular senescence.

Although traditionally known as a cytoskeletal protein, recent research has detected Nestin in the nucleus of various cell types^45–47^. However, the discovery of its involvement in transcriptional regulation is unprecedented. In this report, we reveal a completely new function for Nestin: directly binding to a specific *cis*-RE in the *CDKN2A/B* locus (site S1606) to repress p16^INK4a^ transcription. This is the first evidence that Nestin can influence gene expression at the chromatin level. Although Nestin downregulation was reported to induce senescence by destabilizing lamin-A/C^48^, our study revealed a novel mechanism in that Nestin regulated p16^INK4a^ expression by binding to *cis*-RE S1606, positioning Nestin as a transcriptional regulator in PAEC senescence.

Notably, we observed that SOX17 and Nestin expression decreased with increasing cellular passage, thereby suggesting their involvement in replicative senescence. This finding has important implications for age-related PAH susceptibility. Although idiopathic PAH has historically been considered a disease of younger adults, recent epidemiological studies show increased PAH prevalence and worse outcomes in older individuals^4,49–51^. The passage-dependent decline in SOX17 and Nestin expression may create conditions for enhanced endothelial senescence by upregulating p16^INK4a^, thus contributing to compromised vascular function in older populations. These findings may help explain why aging is an independent risk factor for PAH developmen^49,52^ and suggest that preserving the SOX17–Nestin regulatory axis could benefit older PAH patients.

In conclusion, we demonstrate that reduced SOX17 expression may lead to Nestin downregulation, increased p16^INK4a^ level, and enhanced EC senescence. Targeting the SOX17–Nestin–p16^INK4a^ signaling pathway may be a new therapeutic strategy for PAH.

## Materials and Methods

### Cell Culture and Treatments

Primary pulmonary arterial ECs (PAECs) (WN-10982) were obtained from Warner (Wuhan, China). Cells were cultured at 37°C in 5% CO_2_ in endothelial growth medium-2 (EGM-2) supplemented with 10% fetal bovine serum (FBS). Cells at passages 3 to 15 were used for experiments. PAECs were treated with interleukin-1β (IL-1β) (201-LB-005, R&D Systems) at 10 ng/mL for 24 h. For viral infection, PAECs at 60% confluence were transduced with lentivirus (Shanghai Genechem) and incubated for 24 h. After viral removal, cells were cultured in complete growth medium for an additional 24 h, followed by puromycin selection to establish stable cell lines. Transient knockdown of Nestin in PAECs involved using siRNA (RiboBio) according to the manufacturer’s protocol. All cultures were regularly tested to ensure that they were mycoplasma-free.

### Primers and Antibodies

All primers used in the study were synthesized by Sangon Biotech (Shanghai) and are listed in Supplementary Table 1. The antibodies used are detailed in Supplementary Table 2.

### Western Blot Analysis

Whole-cell lysates were prepared with radioimmunoprecipitation assay buffer (P0013B; Beyotime Institute of Biotechnology) containing protease and phosphatase inhibitors (P1045, Beyotime Institute of Biotechnology). Proteins were resolved on SDS-PAGE and transferred to polyvinylidene fluoride membranes (IPVH00010, Millipore). After blocking with 5% non-fat milk for 1 h, membranes were incubated overnight at 4°C with primary antibodies, then treated with horseradish peroxidase-conjugated secondary antibodies (ZB-2305/ZB-2301, ZSGB-Bio) for 1 h and visualized by using a Bio-Rad gel documentation system. Data are presented as the mean of three independent experiments (n=3).

### Quantitative Real-Time PCR (qRT-PCR)

Total RNA was extracted by using AG RNAex Pro Reagent (AG21102, Accurate Biology). cDNA was synthesized by using the Evo M-MLV Mix Kit (AG11728, Accurate Biology), and qRT-PCR involved the StepOne real-time PCR system with the SYBR Green Premix Pro Taq HS qPCR Kit (AG11718, Accurate Biology) and TaqMan Universal PCR Master Mix (Applied Biosystems). TaqMan probe/primer sets used include p14 (Hs99999189_m1), p15 (Hs00793225_m1), p16 (Hs02902543_m1), ANRIL (Hs04259472_m1), and GAPDH (Hs02786624_g1) as the internal control. Data represent the mean of three independent experiments (n=6).

### Flanking Restriction Enhanced DNA Pulldown-Mass Spectrometry (FREP-MS)

FREP-MS assay was performed as previously described^18,53^. Briefly, the FREP construct DNA S1606 and negative control (approximately 10 μg each) were conjugated to 150 μl streptavidin-coupled Dynabeads. The DNA–bead complexes were incubated with 1 mg nuclear extract from human ECs for 1 h at room temperature. After washing, the complex was sequentially digested with EcoR I (100 units/μl) for 30 min and BamH I (100 units/μl) for 45 min at 37°C to release the *cis*-RE bound proteins. The supernatant was collected for protein identification by mass spectrometry. Proteins detected exclusively in samples but not in controls were identified as *cis*-RE–binding proteins.

### DNA Pull-Down and Western Blot Assay

The DNA pull-down assay involved using a biotinylated 35-bp DNA fragment generated from annealed primers (IDT). The DNA sample (1 μg) was conjugated to Dynabeads M-280 Streptavidin and incubated with 100 μg PAEC nuclear extract for 1 h at room temperature. After washing, DNA-bound proteins were eluted and analyzed by SDS-PAGE, followed by western blot analysis with specific antibodies. Data represent three independent experiments (n=3).

### Luciferase Reporter Assay

Luciferase reporter assays involved HEK293T cells transfected with pGL3-promoter vectors containing S1606 and TTCCAGGC sequences from the Nestin promoter. Transfections involved using FuGENE HD reagent (E2311, Promega). Luciferase activity was measured with the Dual-Glo Luciferase Reporter Assay System (E2920, Promega). All assays were conducted in triplicate (n=6).

### Chromatin Immunoprecipitation (ChIP) Assay

ChIP assays involved the Pierce Magnetic ChIP Kit (26157; Thermo Scientific) following the manufacturer’s instructions. Cells were crosslinked with 1% formaldehyde, then sonicated at 30% amplitude with 20 s “on” and 50 s “off” intervals for a total of 5 min. Sonicated chromatin was incubated overnight with 10 μg gene-specific antibodies coupled to Dynabeads Protein A/G. After reversing the crosslink, purified DNA was used for qRT-PCR. Rabbit IgG was used as a control. Data represent three independent experiments (n = 3).

### Senescence-associated β-galactosidase (SA-β-Gal) Staining

SA-β-gal staining involved using the manufacturer’s protocol (CST9860S, Cell Signaling Technology). Cells were fixed, washed, and stained with SA-β-gal solution overnight at 37°C. Images were captured under an RVL-100-G microscope (Echo Laboratories) and were analyzed by using ImageJ. Data are representative of three independent experiments (n = 3).

### γ-H2AX Staining

Cells were fixed in 4% paraformaldehyde for 15 min, permeabilized, and incubated with γ-H2AX antibody (sc-517348, Santa Cruz Biotechnology) overnight at 4°C. Secondary antibodies conjugated with Alexa Fluor 488 (A28175, Invitrogen) were applied, followed by DAPI counterstaining. Cells were imaged, and data were analyzed by using ImageJ. Results represent three independent experiments (n = 3).

### Animal PAH Model and AAV Gene Transfer

All animal procedures were approved by the Animal Care and Use Committee of Xiangya Hospital, China, and conducted in accordance with NIH guidelines. Four-week-old male Sprague-Dawley rats were injected intraperitoneally with 60 mg/kg monocrotaline (MCT) (C2401, Sigma-Aldrich). AAV serotype 9 (AAV9) encoding rat SOX17 (1×10^13^ vg/mL) or Nestin (1×10^13^ vg/mL) was administered intratracheally 3 weeks before MCT treatment. Hemodynamic measurements and histological assessments were conducted 4 weeks post-MCT administration.

### Human samples

PAH was defined by elevated mean pulmonary arterial pressure (mPAP) ≥ 20 mmHg. Lung samples were collected from discarded surgical samples or rapid autopsy samples from individuals with a diagnosis of PAH (Supplemental Table 3). Non-diseased lung specimens were from the Center for Organ Recovery & Education (CORE; Pittsburgh, PA, USA).

### Histology and Immunofluorescence Staining

Lung tissue sections (4-μm) were prepared and underwent hematoxylin-eosin staining. For immunofluorescence staining, antigen retrieval was followed by blocking in 5% goat serum. Sections were incubated with primary antibodies overnight, followed by secondary antibodies conjugated to Alexa Fluor 488 or 594. The extent of pulmonary arteriolar medial thickening was assessed by calculating the proportion of fully versus partially muscularized arterioles stained with α-smooth muscle actin.

### Primary Pulmonary Vascular EC Isolation

Pulmonary vascular ECs were isolated as described previously^54^. Briefly, lung tissue was digested with collagenase, and cells were separated by centrifugation. ECs were purified by using the Miltenyi Biotec EC isolation kit (130–109-690) following the manufacturer’s protocol.

### Microarray data

GSE154959 profile was selected from the Gene Expression Omnibus (GEO) database (http://www.ncbi.nlm.nih.gov/geo/). The GSE154959 dataset, including one control mice, and three mice exposed to SU5416 and hypoxia (SuHx), is based on the GPL24247 platform (Illumina NovaSeq 6000). Bioinformatics analysis involved using R (https://www.r-project.org/). Expression patterns of specific genes (SOX17, Nestin, and p16^INK4a^) were visualized as dot plots, with dot size representing the proportion of cells expressing each gene and color intensity the average expression level. Gene Ontology Biological Process (GO-BP) enrichment analysis involved using clusterProfiler with a focus on cellular senescence pathways. Enrichment scores were compared between normoxic and hypoxic conditions.

### Statistical Analysis

Data are presented as mean and standard error of the mean (SEM). P-values were calculated with two-tailed Student *t* test or non-parametric tests as appropriate. All analyses were conducted with ImageJ 1.53a, SPSS 26, or GraphPad Prism 8. Statistical significance was set at p<0.05.

## Supplementary Files

This is a list of supplementary files associated with this preprint. Click to download.

- Supplementarymaterials06.11.docx

## References

[1] Simonneau G., Haemodynamic definitions and updated clinical classification of pulmonary hypertension. Eur Respir J 53(2019). 10.1183/13993003.01913-2018 PMC6351336 30545968

[2] Thenappan T., Ormiston M.L., Ryan J.J. & Archer S.L. Pulmonary arterial hypertension: pathogenesis and clinical management. Bmj 360, j5492 (2018). 29540357 10.1136/bmj.j5492 PMC6889979

[3] D’Alonzo G.E., Survival in patients with primary pulmonary hypertension. Results from a national prospective registry. Ann Intern Med 115, 343–349 (1991). 1863023 10.7326/0003-4819-115-5-343

[4] Hoeper M.M., A global view of pulmonary hypertension. Lancet Respir Med 4, 306–322 (2016). 26975810 10.1016/S2213-2600(15)00543-3

[5] Benza R.L., An evaluation of long-term survival from time of diagnosis in pulmonary arterial hypertension from the REVEAL Registry. Chest 142, 448–456 (2012). 22281797 10.1378/chest.11-1460

[6] Bian J.S., ErbB3 Governs Endothelial Dysfunction in Hypoxia-Induced Pulmonary Hypertension. Circulation 150, 1533–1553 (2024). 38214194 10.1161/CIRCULATIONAHA.123.067005

[7] Shen Y.H., Panorama of artery endothelial cell dysfunction in pulmonary arterial hypertension. J Mol Cell Cardiol (2024). 10.1016/j.yjmcc.2024.10.004 39437884

[8] Zou X., SOX17 Prevents Endothelial-Mesenchymal Transition of Pulmonary Arterial Endothelial Cells in Pulmonary Hypertension through Mediating TGF-β/Smad2/3 Signaling. Am J Respir Cell Mol Biol (2024). 10.1165/rcmb.2023-0355OC 39392679

[9] Liu B., General Capillary Endothelial Cells Undergo Reprogramming Into Arterial Endothelial Cells in Pulmonary Hypertension Through HIF-2α/Notch4 Pathway. Circulation 150, 414–417 (2024). 39074180 10.1161/CIRCULATIONAHA.123.067981 PMC11296501

[10] Culley M.K., Frataxin deficiency promotes endothelial senescence in pulmonary hypertension. J Clin Invest 131(2021). 10.1172/JCI136459 PMC8159699 33905372

[11] Culley M.K. & Chan S.Y. Endothelial Senescence: A New Age in Pulmonary Hypertension. Circ Res 130, 928–941 (2022). 35298304 10.1161/CIRCRESAHA.121.319815 PMC8943914

[12] van der Feen D.E., Cellular senescence impairs the reversibility of pulmonary arterial hypertension. Sci Transl Med 12(2020). 10.1126/scitranslmed.aaw4974 PMC7891555 32727916

[13] Bloom S.I., Islam M.T., Lesniewski L.A. & Donato A.J. Mechanisms and consequences of endothelial cell senescence. Nat Rev Cardiol 20, 38–51 (2023). 35853997 10.1038/s41569-022-00739-0 PMC10026597

[14] Ramadhiani R., Endothelial cell senescence exacerbates pulmonary hypertension by inducing juxtacrine Notch signaling in smooth muscle cells. iScience 26, 106662 (2023). 37192975 10.1016/j.isci.2023.106662 PMC10182325

[15] Hannou S.A., Wouters K., Paumelle R. & Staels B. Functional genomics of the CDKN2A/B locus in cardiovascular and metabolic disease: what have we learned from GWASs? Trends Endocrinol Metab 26, 176–184 (2015). 25744911 10.1016/j.tem.2015.01.008

[16] Jiang D., Post-GWAS functional analysis identifies CUX1 as a regulator of p16(INK4a) and cellular senescence. Nat Aging 2, 140–154 (2022). 37117763 10.1038/s43587-022-00177-0 PMC10154215

[17] Hernandez-Segura A., Nehme J. & Demaria M. Hallmarks of Cellular Senescence. Trends Cell Biol 28, 436–453 (2018). 29477613 10.1016/j.tcb.2018.02.001

[18] Wu T., Coupling high-throughput mapping with proteomics analysis delineates *cis*-regulatory elements at high resolution. Nucleic Acids Res 50, e5 (2022). 34634809 10.1093/nar/gkab890 PMC8754656

[19] Baker D.J., Clearance of p16Ink4a-positive senescent cells delays ageing-associated disorders. Nature 479, 232–236 (2011). 22048312 10.1038/nature10600 PMC3468323

[20] Baker D.J., Naturally occurring p16(Ink4a)-positive cells shorten healthy lifespan. Nature 530, 184–189 (2016). 26840489 10.1038/nature16932 PMC4845101

[21] Lendahl U., Zimmerman L.B. & McKay R.D. CNS stem cells express a new class of intermediate filament protein. Cell 60, 585–595 (1990). 1689217 10.1016/0092-8674(90)90662-x

[22] Park D., Nestin is required for the proper self-renewal of neural stem cells. Stem Cells 28, 2162–2171 (2010). 20963821 10.1002/stem.541

[23] Saboor F., Nestin-expressing vascular wall cells drive development of pulmonary hypertension. Eur Respir J 47, 876–888 (2016). 26699726 10.1183/13993003.00574-2015 PMC5796529

[24] Bhagwani A.R., Endothelial cells are a source of Nestin expression in Pulmonary Arterial Hypertension. PLoS One 14, e0213890 (2019). 30883593 10.1371/journal.pone.0213890 PMC6422269

[25] Zhou J.J., Nestin represents a potential marker of pulmonary vascular remodeling in pulmonary arterial hypertension associated with congenital heart disease. J Mol Cell Cardiol 149, 41–53 (2020). 32950539 10.1016/j.yjmcc.2020.09.005

[26] Francois M., Koopman P. & Beltrame M. SoxF genes: Key players in the development of the cardiovascular system. Int J Biochem Cell Biol 42, 445–448 (2010). 19733255 10.1016/j.biocel.2009.08.017

[27] Zhu N., Rare variants in SOX17 are associated with pulmonary arterial hypertension with congenital heart disease. Genome Med 10, 56 (2018). 30029678 10.1186/s13073-018-0566-x PMC6054746

[28] Park C.S., Sox17 Deficiency Promotes Pulmonary Arterial Hypertension via HGF/c-Met Signaling. Circ Res 131, 792–806 (2022). 36205124 10.1161/CIRCRESAHA.122.320845 PMC9612711

[29] Zou X., SOX17 is a Critical Factor in Maintaining Endothelial Function in Pulmonary Hypertension by an Exosome-Mediated Autocrine Manner. Adv Sci (Weinh) 10, e2206139 (2023). 36919784 10.1002/advs.202206139 PMC10190640

[30] Sangam S., SOX17 Deficiency Mediates Pulmonary Hypertension: At the Crossroads of Sex, Metabolism, and Genetics. Am J Respir Crit Care Med 207, 1055–1069 (2023). 36913491 10.1164/rccm.202203-0450OC PMC10112457

[31] González-Hernández S., Sox17 Controls Emergence and Remodeling of Nestin-Expressing Coronary Vessels. Circ Res 127, e252–e270 (2020). 32921258 10.1161/CIRCRESAHA.120.317121

[32] Grosse L., Defined p16(High) Senescent Cell Types Are Indispensable for Mouse Healthspan. Cell Metab 32, 87–99.e86 (2020). 32485135 10.1016/j.cmet.2020.05.002

[33] Coppé J.P., Tumor suppressor and aging biomarker p16(INK4a) induces cellular senescence without the associated inflammatory secretory phenotype. J Biol Chem 286, 36396–36403 (2011). 21880712 10.1074/jbc.M111.257071 PMC3196093

[34] Rodor J., Single-cell RNA sequencing profiling of mouse endothelial cells in response to pulmonary arterial hypertension. Cardiovasc Res 118, 2519–2534 (2022). 34528097 10.1093/cvr/cvab296 PMC9400412

[35] Yi D., E2F1 Mediates SOX17 Deficiency-Induced Pulmonary Hypertension. Hypertension 80, 2357–2371 (2023). 37737027 10.1161/HYPERTENSIONAHA.123.21241 PMC10591929

[36] Born E., Eliminating Senescent Cells Can Promote Pulmonary Hypertension Development and Progression. Circulation 147, 650–666 (2023). 36515093 10.1161/CIRCULATIONAHA.122.058794

[37] Kyi P., Endothelial senescence mediates hypoxia-induced vascular remodeling by modulating PDGFB expression. Front Med (Lausanne) 9, 908639 (2022). 36203755 10.3389/fmed.2022.908639 PMC9530050

[38] Rhodes C.J., Genetic determinants of risk in pulmonary arterial hypertension: international genome-wide association studies and meta-analysis. Lancet Respir Med 7, 227–238 (2019). 30527956 10.1016/S2213-2600(18)30409-0 PMC6391516

[39] Gräf S., Identification of rare sequence variation underlying heritable pulmonary arterial hypertension. Nat Commun 9, 1416 (2018). 29650961 10.1038/s41467-018-03672-4 PMC5897357

[40] Wu Y., The pathophysiological role of novel pulmonary arterial hypertension gene SOX17. Eur Respir J 58(2021). 10.1183/13993003.04172-2020 33632800

[41] Liu M., Sox17 is required for endothelial regeneration following inflammation-induced vascular injury. Nat Commun 10, 2126 (2019). 31073164 10.1038/s41467-019-10134-y PMC6509327

[42] Walters R., SOX17 Enhancer Variants Disrupt Transcription Factor Binding And Enhancer Inactivity Drives Pulmonary Hypertension. Circulation 147, 1606–1621 (2023). 37066790 10.1161/CIRCULATIONAHA.122.061940 PMC7614572

[43] Ainscough A.J., An organ-on-chip model of pulmonary arterial hypertension identifies a BMPR2-SOX17-prostacyclin signalling axis. Commun Biol 5, 1192 (2022). 36344664 10.1038/s42003-022-04169-z PMC9640600

[44] Zou X., SOX17 Prevents Endothelial-Mesenchymal Transition of Pulmonary Arterial Endothelial Cells in Pulmonary Hypertension through Mediating TGF-β/Smad2/3 Signaling. Am J Respir Cell Mol Biol 72, 364–379 (2025). 39392679 10.1165/rcmb.2023-0355OC

[45] Loja T., Characterization of a GM7 glioblastoma cell line showing CD133 positivity and both cytoplasmic and nuclear localization of nestin. Oncol Rep 21, 119–127 (2009). 19082452

[46] Thomas S.K., Messam C.A., Spengler B.A., Biedler J.L. & Ross R.A. Nestin is a potential mediator of malignancy in human neuroblastoma cells. J Biol Chem 279, 27994–27999 (2004). 15117961 10.1074/jbc.M312663200

[47] Chen Z., Role of the stem cell-associated intermediate filament nestin in malignant proliferation of non-small cell lung cancer. PLoS One 9, e85584 (2014). 24498263 10.1371/journal.pone.0085584 PMC3911905

[48] Zhang Y., Nuclear Nestin deficiency drives tumor senescence via lamin A/C-dependent nuclear deformation. Nat Commun 9, 3613 (2018). 30190500 10.1038/s41467-018-05808-y PMC6127343

[49] Lam C.S., Age-associated increases in pulmonary artery systolic pressure in the general population. Circulation 119, 2663–2670 (2009). 19433755 10.1161/CIRCULATIONAHA.108.838698 PMC2753443

[50] Ling Y., Changing demographics, epidemiology, and survival of incident pulmonary arterial hypertension: results from the pulmonary hypertension registry of the United Kingdom and Ireland. Am J Respir Crit Care Med 186, 790–796 (2012). 22798320 10.1164/rccm.201203-0383OC

[51] Rose J.A., Cleveland J.M., Rao Y., Minai O.A. & Tonelli A.R. Effect of Age on Phenotype and Outcomes in Pulmonary Arterial Hypertension Trials. Chest 149, 1234–1244 (2016). 26836910 10.1016/j.chest.2015.11.008 PMC4944788

[52] Hjalmarsson C., Impact of age and comorbidity on risk stratification in idiopathic pulmonary arterial hypertension. Eur Respir J 51(2018). 10.1183/13993003.02310-2017 29622568

[53] Li G., High-throughput identification of noncoding functional SNPs via type IIS enzyme restriction. Nat Genet 50, 1180–1188 (2018). 30013183 10.1038/s41588-018-0159-z PMC6072570

[54] Zhang C., Mitomycin C induces pulmonary vascular endothelial-to-mesenchymal transition and pulmonary veno-occlusive disease via Smad3-dependent pathway in rats. Br J Pharmacol 178, 217–235 (2021). 33140842 10.1111/bph.15314

